# Retinal Microvascular Changes in Association with Endothelial Glycocalyx Damage and Arterial Stiffness in Patients with Diabetes Mellitus Type 2: A Cross-Sectional Study in a Greek Population

**DOI:** 10.3390/jpm14090995

**Published:** 2024-09-19

**Authors:** Chrysa Agapitou, Theodoros N. Sergentanis, John Thymis, George Pavlidis, Stamatios Lampsas, Emmanouil Korakas, Aikaterini Kountouri, Loukia Pliouta, Efthymios Karmiris, Areti Lagiou, Panagiotis Theodossiadis, Vaia Lambadiari, Ignatios Ikonomidis, Irini Chatziralli

**Affiliations:** 1 2nd Department of Ophthalmology, Attikon Hospital, Medical School, National and Kapodistrian University of Athens, 124 62 Athens, Greece; 2 Department of Public Health Policy, School of Public Health, University of West Attica, 115 21 Athens, Greece; 3 2nd Department of Cardiology, Attikon Hospital, Medical School, National and Kapodistrian University of Athens, 124 62 Athens, Greece; ioanth@med.uoa.gr (J.T.); geo_pavlidis@yahoo.gr (G.P.);; 4 Research Unit and Diabetes Centre, 2nd Department of Internal Medicine, Attikon Hospital, Medical School, National and Kapodistrian University of Athens, 124 62 Athens, Greece; 5 Laboratory of Hygiene and Epidemiology, Department of Public and Community Health, University of West Attica, 124 62 Athens, Greece

**Keywords:** diabetic, OCT-A, microvascular, endothelial dysfunction, arterial stiffness

## Abstract

Purpose: To evaluate the potential association between endothelial glycocalyx damage, as well as arterial stiffness, and the retinal changes on optical coherence tomography (OCT) and OCT-angiography (OCT-A) in patients with type 2 diabetes mellitus (DM). Methods: Participants in this cross-sectional study were 65 patients with DM type 2 and 42 age- and gender-matched controls without DM. The demographic and clinical characteristics of the participants were recorded. All patients underwent a thorough ophthalmological examination and multimodal imaging, including fundus photography, OCT, and OCT-A. In addition, evaluation of the endothelial glycocalyx thickness by measuring the perfused boundary region (PBR5-25) of the sublingual microvessel, as well as of the arterial stiffness, by measuring the carotid–femoral pulse wave velocity (PWV), the central aortic pressures and the augmentation index (Aix) was performed. Univariate and multivariate logistic regression analysis was performed for the examination of the potential association between the eye imaging variables and the cardiovascular-related variables. The odds ratios (OR) with the respective 95% confidence intervals (CI) were calculated. A *p*-value < 0.05 was considered statistically significant. Results: Patients with DM presented significantly higher PBR5-25 compared to controls without DM (*p* = 0.023). At the univariate analysis, increased PBR5-25 (≥2.19 μm vs. <2.19 μm) was associated with decreased peripapillary VD at the superior quadrant (univariate OR (95% CI) = 0.34 (0.12–0.93), *p* = 0.037). Multivariate logistic regression analysis showed that increased PWV (≥13.7 m/s vs. <13.7 m/s) was associated with an increased foveal avascular zone (FAZ) area on OCT-A (*p* = 0.044) and increased FAZ perimeter (*p* = 0.048). Moreover, increased Aix (≥14.745% vs. <14.745%) was associated with diabetic macular edema (DME) presence (*p* = 0.050) and increased perifoveal and parafoveal superior and temporal thickness on OCT (*p* < 0.05 for all associations). Conclusions: Markers of endothelial damage and arterial stiffness were associated with structural and microvascular retinal alterations in patients with DM, pointing out that OCT-A could be a useful biomarker for detecting potential cardiovascular risk in such patients.

## 1. Introduction

Diabetes mellitus (DM) is a global health issue, affecting approximately 540 million adults in 2021, while it is expected to reach about 785 million by 2045 [1,2]. The increasing prevalence of diabetes has resulted in an increase in complications, including diabetic retinopathy (DR) [1]. DR is a common microvascular complication of DM, affecting around one-third of people with DM globally, and it is the leading cause of visual impairment among the working-age population [2]. DR is classified as non-proliferative (NPDR) and proliferative (PDR). NPDR is characterized by structural changes in retinal capillaries, including microaneurysms, hemorrhages, exudates, cotton-wool spots, venous beading or looping, intraretinal microvascular abnormalities (IRMA), and retinal non-perfusion. The latter may trigger the development of neovascularization either on the disc (NVD) or elsewhere on the retina (NVE), leading to PDR [3]. It is worth noting that diabetic macular edema (DME) may occur at any stage of DR [3].

In the pathogenesis of DR, chronic hyperglycemia leads to structural changes in the retina vessels’ wall through several biochemical pathways based on oxidative stress. These changes include the thickening of the capillaries’ basement membrane, causing hypoxia and pericyte loss, resulting in endothelial damage and consequently, disruption of the inner blood–retina barrier [4,5,6]. Moreover, local inflammation has been also implicated in the pathophysiology of DR, since elevated levels of pro-inflammatory mediators, such as interleukin-6 (IL-6), interleukin-8 (IL-8), tumor necrosis factor-a (TNF-a), monocyte chemoattractant protein-1 (MCP-1), intracellular adhesion molecule-1 (ICAM-1), and vascular endothelial growth factor (VEGF) have been found elevated in aqueous or vitreous fluids of patients with DR [7,8].

It is worth noting that patients with DM type 2 have an increased risk for cardiovascular diseases, macrovascular events, and mortality [9]. Endothelial dysfunction and arterial stiffness are significant factors in cardiovascular pathophysiology and seem to be also implicated in DR pathogenesis [10,11,12]. Of note, DR and arterial stiffness are interconnected complications commonly seen in patients with DM, with both reflecting the systemic vascular damage induced by chronic hyperglycemia and sharing similar pathophysiological mechanisms [3]. Specifically, persistent hyperglycemia contributes to endothelial dysfunction and the formation of advanced glycation end-products (AGEs), which accumulate in the arterial walls and the retina, promoting vascular stiffness and microvascular damage [13], while endothelial dysfunction also impairs vasodilation, increases vascular permeability, and promotes atherosclerosis [14]. An et al. have shown that brachial–ankle pulse wave velocity (baPWV), an indicator of arterial stiffness, might be an independent predictor in the new onset or worsening of DR, suggesting that increased arterial stiffness might be involved in the development of DR [11]. Moreover, an increasing degree of PWV has been found to be positively associated with the severity of DR; high PWV was strongly associated with the risk of severe DR, especially PDR [15]. Indeed, DR is a microvascular complication, while arterial stiffness is indicative of macrovascular disease. The coexistence of these complications highlights the systemic nature of vascular damage in DM, emphasizing the need for comprehensive cardiovascular risk management in diabetic patients [16].

Furthermore, patients with DM have been demonstrated to present microvascular changes on optical coherence tomography–angiography (OCT-A), such as foveal avascular zone (FAZ) enlargement, increased capillary non-perfusion, and decreased vessel density [17,18,19,20,21]. Of note, previous studies have shown that retinal neurovascular changes seen in OCT and OCT-A might mimic systemic arterial stiffness in patients with DM type 2 [22], while greater carotid stiffness has been associated with worse retinal flicker light-induced dilation [23]. Moreover, Kim et al. found that there was a significant correlation between arteriosclerosis and choroidal vascular changes in DR [24]. However, there are no studies examining endothelial glycocalyx integrity in association with OCT-A findings in patients with DM type 2.

In light of the above, the purpose of this study was to evaluate the potential association between endothelial damage, as well as arterial stiffness, and retinal changes on OCT and OCT-A in Greek patients with DM type 2.

## 2. Methods

The participants in this cross-sectional study were 65 patients with DM type 2 diagnosed at the 2nd Department of Ophthalmology, National and Kapodistrian University of Athens, Athens, Greece, between 1 March 2024 and 31 May 2024. In addition, 42 controls without DM, age- and gender-matched to patients, were included in the study. Patients with media opacities, uveitis, uncontrolled glaucoma with intraocular pressure (IOP) ≥ 30 mmHg, trauma, and any previous intraocular surgery during the last 6 months were excluded from the study.

The study was conducted in accordance with the Declaration of Helsinki and approved by the Institutional Review Board of Attikon University Hospital (reference number 324/2024 and approval date 17 January 2024). Informed consent was obtained from all subjects involved in the study. 

The demographic and clinical characteristics of the participants were recorded. All patients underwent a complete ophthalmologic examination, including best-corrected visual acuity (BCVA) measurement by means of Snellen charts, slit-lamp examination, dilated fundoscopy, color fundus photography, swept source-OCT (SS-OCT) and OCT-A, using Topcon DRI OCT Triton Plus (Topcon, Tokyo, Japan). The machine uses an angiography ratio analysis (OCTARA) algorithm. It has an A-scan rate of 100,000 A-scans per second and works with a long wavelength scanning light of 1050 nm. It has an axial resolution of 8 µm, a lateral resolution of 20 µm, and an imaging depth of 2.6 mm [25].

In all patients, we assessed diabetic retinopathy (DR) severity and graded it as no DR, mild non-proliferative DR (NPDR), moderate NPDR, and severe NPDR and PDR, while the presence of diabetic macular edema was also evaluated.

Regarding the SS-OCT protocol, the scans were performed to obtain measurements of the macular area and peripapillary retinal nerve fiber layer (RNFL), using the macular protocol 3D 7 × 7 mm (H) and the peripapillary protocol 3D Disc 6 × 6 mm. The 3D(H) Macular protocol performs a 7.0 × 7.0 mm 3D scan of the macular area, providing measurements of the 9 macular areas of the Early Treatment Diabetic Retinopathy Study (ETDRS scan). With the nine ETDRS macular areas (which include a central 1 mm circle representing the fovea and inner and outer rings measuring 3 and 6 mm in diameter), full retinal thickness is measured. In the peripapillary area, measurements of the RNFL thickness of four quadrants (superior, nasal, inferior, and temporal) as well as average RNFL thickness were analyzed, using the 3D Disc 6 × 6 mm protocol. All scans were performed by the same experienced operator (CA).

**Figure 1:**
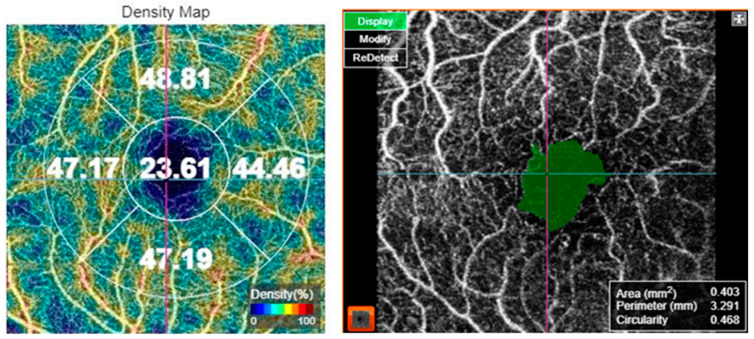
Optical coherence tomography angiography of a 69-year-old female patient with diabetes mellitus type 2, showing the vessel density map (**left**) and the foveal avascular zone metrics (**right**).

For OCT-A acquisition, the 3 × 3 mm protocol for macular OCT-A. The scan was verified for foveal centration. In cases with decentration, the early treatment of diabetic retinopathy study (ETDRS) grid was centered on the fovea manually. The superficial capillary plexus was segmented by the automated software. Macular vascular density (MVD) was measured by the manufacturer’s software in the superficial capillary plexus slab. MVD was measured in five regions: central, inner superior, inner nasal, inner inferior, and inner temporal subfields of the ETDRS grid. Moreover, FAZ metrics including FAZ area, FAZ perimeter, and FAZ circularity index were assessed, as shown in Figure 1. Optic nerve head angiography was also performed using the 4.5 × 4.5 mm protocol. The scan was centered on the optic disc. All images were captured by the same investigator (CA). The OCT-A images were analyzed for the presence of artifacts and in such cases, the images were captured again. Only images with a scan quality score > 55 were included in the analysis; poor-quality images prior to data analysis were rejected by the operator and the scan was repeated until good quality was achieved.

Apart from ocular examination and retinal imaging, all patients underwent evaluation of the endothelial function by measuring the endothelial glycocalyx thickness. We indirectly assessed endothelial glycocalyx integrity by measuring the perfused boundary region (PBR), which is the erythrocyte-penetrable area of the endothelial glycocalyx. The PBR is the cell-poor layer that results from the separation between the flowing red blood cell column and plasma on the surface of the vascular lumen. A larger PBR indicates a deeper penetration of erythrocytes into the glycocalyx, which corresponds with greater endothelial glycocalyx impairment [26,27,28,29]. Hence, PBR is inversely proportional to EG thickness. Specifically, the PBR of the sublingual arterial microvessels with a diameter that ranged from 5 to 25 μm was measured using Sidestream Dark Field imaging (Microscan, Glycocheck, Microvascular Health Solutions Inc, Salt Lake City, UT, USA). This technique provides a fast and noninvasive assessment of the endothelial glycocalyx thickness, as previously described [27]. The assessment of glycocalyx thickness using dedicated cameras provides measurements of multiple sample sites (>3000 vascular segments of sublingual microvessels) within 3 min and has good reproducibility [27,28]. Therefore, this technique was proposed as a valid technique to assess endothelial integrity by the European Society of Cardiology Working Group on Peripheral Circulation [29].

Moreover, we measured the carotid–femoral PWV, augmentation index (Aix), and central aortic pressures (central systolic and diastolic-cSBP and cDBP) using tonometry by a Complior (Alam Medical, Vincennes, France). Normal values were PWV < 10 m/s [30,31]. Aix was defined as 100 × (P2 − P1)/PP, where P2 is the late backward systolic wave, P1 is the early forward systolic wave, and PP is the pulse pressure and represents the pressure boost that is induced by the return of the reflected waves at the aorta [31].

### Statistical Analysis

For the statistical analysis, we included one eye per patient. In all of the cases, the right eye was chosen to avoid selection bias. For the description of patients’ characteristics, descriptive statistics were calculated. The mean ± standard deviation (SD) was used for continuous variables, which were normally distributed; for variables with skewed distribution, we used median and interquartile range (IQR) instead. Relative frequencies and percentages for categorical variables were reported. The Kolmogorov–Smirnov and the Shapiro–Wilk normality tests were applied for data distribution assessment.

For the comparison regarding PBR5-25 between patients and controls, the Mann–Whitney–Wilcoxon test was used.

Regarding the logistic regression analyses, an a priori conceptual framework was followed. At the univariate models, logistic regression analysis was performed for the examination of the potential associations between the eye imaging variables (central subfield thickness, perifoveal and parafoveal macular thickness in 4 quadrants on OCT; average RNFL thickness and RNFL thickness in 4 quadrants on optic nerve OCT; FAZ area, FAZ perimeter, FAZ circularity, central VD and VD at the 4 macular quadrants on macular OCT-A; peripapillary VD at the 4 quadrants on OCT-A of the optic nerve), which were treated as dependent variables, and each cardiovascular-related variable (PBR; PWV; Aix), which were the independent variables (binary variables, ≥median vs. <median) in separate analysis for each independent variable. Subsequently, in the multivariate logistic regression analysis, the same structure was followed regarding the dependent and independent variables, but adjustment for gender, age (≥median vs. <median), and DM duration (≥median vs. <median) was performed. Once again, a separate series of models was conducted for i. PBR; ii. PWV and iii. Aix as independent variables. The odds ratios with the respective 95% confidence intervals are indicated in the text. Statistical analysis was performed using STATA/SE 13.0 statistical software (Stata Corporation, College Station, TX, USA). A *p*-value < 0.05 was considered statistically significant.

## 3. Results

The participants in the study were 65 patients with DM type 2. Table 1 shows the demographic and clinical characteristics of our study sample. The mean age of patients was 66.6 ± 8.7 years. Overall, 38 out of 65 patients (58.5%) were male and 27 (41.5%) were female. Regarding comorbidities, 48 out of 65 patients (73.8%) had hypertension, 52 (80%) had dyslipidemia, 6 (9.2%) had nephropathy, 15 (23.1%) had neuropathy, and 11 (16.9%) had cardiovascular disorders. The mean duration of DM was 16.4 ± 11.2 years and the mean HbA1c was 7.2 ± 1.1%. In total, 28 out of 65 patients (43.1%) had DME, while 30.8% of patients had no DR, 4.6% had mild NPDR, 21.5% had moderate NPDR, 35.4% had severe NPDR, and 7.7% had PDR. The mean visual acuity was the 0.44 ± 0.19 decimal scale. The mean intraocular pressure was 13.4 ± 3.1 mmHg. Overall, 64.6% of patients were phakic and 35.4% were pseudophakic. 

In addition, 42 subjects without DM were used as controls for comparison with patients with DM regarding the values of PBR. The mean age of controls was 66.5 ± 8.6 years. In total, 23 out of 42 patients (54.8%) were male and 19 (45.2%) female.

The systemic vascular metrics are shown in Table 2. In patients with DM, the mean PBR5-25 was 2.20 ± 0.26 μm. The mean cSBP was 130.4 ± 20.6 mmHg, while the mean cDBP was 77.6 ± 10.1 mmHg. The mean PWV was measured to be 13.5 ± 4.9 m/s and the mean Aix was 16.3 ± 23.6%. Regarding the PBR5-25, there was a statistically significant difference between patients with DM (median (1st–3rd quartile) = 2.19 (2.07–2.36)) and controls (median (1st–3rd quartile) = 2.07 (1.89–2.24)) (*p* = 0.023, Mann–Whitney–Wilcoxon for independent samples).

The measurements for all retinal parameters on OCT and OCT-A were included on Table 3. The mean central subfield thickness was 257.4 ± 36.2 μm. The mean average RNFL thickness was 100.1 ± 12.9 μm. The mean central vessel density (VD) was 18.9 ± 5.5%, while the mean FAZ area was 0.343 ± 0.146 mm^2^ with a FAZ circularity index of 0.560 ± 0.079. Regarding the optic disc OCT-A, the mean average peripapillary VD was 41.5 ± 3.19%.

Table 4 shows the results of a series of logistic regression models, examining the associations between the increased diameter of PBR5-25 (≥2.19 μm versus <2.19 μm) and eye imaging variables, where 2.19 mm is the median value of the PBR5-25. At the univariate analysis, increased PBR5-25 (>=2.19 vs. <2.19) was associated with decreased peripapillary VD at the superior quadrant (univariate OR (95% CI) = 0.34 (0.12–0.93), *p* = 0.037). Specifically, patients with PBR5-25 ≥ 2.19 had a 66% decrease in the odds of a larger peripapillary VD at the superior quadrant (≥47.74%) versus patients with PBR < 2.19. However, this trend lost its significance in the multivariate analysis, but the OR remained sizeable (adjusted OR (95% CI) = 0.37 (0.12–1.12), *p* = 0.079).

Table 5 shows the results of the logistic regression analysis, examining the associations between increased PWV (≥13.7 m/s versus <13.7 m/s) and eye imaging variables, where 13.7 is the median value of the PWV. At the univariate analysis, increased PWV (≥13.7 m/s vs. <13.7 m/s) was associated with increased FAZ area on OCT-A (univariate OR (95% CI) = 2.79 (1.01–7.70), *p* = 0.048) and increased FAZ perimeter (OR (95% CI) = 2.79 (1.01–7.70), *p* = 0.048), as well as decreased VD at the nasal quadrant (OR (95% CI) = 0.36 (0.13–0.99), *p* = 0.048). At the multivariate analysis, increased PWV (≥13.7 m/s vs. <13.7 m/s) was associated with an increased FAZ area on OCT-A (adjusted OR (95% CI) = 2.88 (1.03–8.04), *p* = 0.044) and an increased FAZ perimeter (adjusted OR (95% CI) = 2.82 (1.01–7.89), *p* = 0.048), while the association between PWV and VD at the nasal quadrant lost its significance (*p* = 0.054). Specifically, patients with PWV ≥ 13.7 m/s had a 188% increase in the odds of a larger FAZ area (≥0.304 mm^2^) and a 182% increase in the odds of a larger FAZ perimeter (≥2.601 mm).

Table 6 shows the results of the logistic regression analysis, examining the associations between increased Aix (≥14.745% versus <14.745%) and eye imaging variables, where 14.745 is the median value of the Aix. At the univariate analysis, increased Aix (≥14.745% vs. <14.745%) was associated with increased parafoveal temporal thickness on OCT (univariate OR (95% CI) = 2.78 (1.01–7.64), *p* = 0.048). At the multivariate analysis, increased Aix (≥14.745% vs. <14.745%) was associated with DME presence [adjusted OR (95% CI) = 3.05 (1.00–9.34), *p* = 0.050], increased perifoveal superior thickness on OCT [adjusted OR (95% CI) = 3.37 (1.07–10.60), *p* = 0.038], increased perifoveal temporal thickness on OCT (adjusted OR (95% CI) = 3.37 (1.04–10.90), *p* = 0.043), increased parafoveal superior thickness on OCT (adjusted OR (95% CI) = 3.47 (1.11–10.78), *p* = 0.032), and increased parafoveal temporal thickness on OCT (adjusted OR (95% CI) = 3.41 (1.11–10.49), *p* = 0.032). Specifically, patients with Aix ≥ 14.745% had a 205% increase in the odds of having DME, a 237% increase in the odds of a thicker perifoveal superior quadrant (≥318 μm), a 237% increase in the odds of a thicker perifoveal temporal quadrant (≥322 μm), a 247% increase in the odds of a thicker parafoveal superior quadrant (≥269 μm), and a 241% increase in the odds of a thicker parafoveal temporal quadrant (≥265 μm).

## 4. Discussion

The principal message of this study was that cardiovascular-related parameters (namely PBR5-25, PWV, and Aix) were associated with eye imaging findings on OCT and OCT-A. Specifically, patients with PBR5-25 ≥ 2.19μm had decreased odds of a larger peripapillary VD at the superior quadrant, although this finding lost its significance at the multivariate analysis. Furthermore, patients with PWV ≥ 13.7 m/s had increased odds of a larger FAZ area and a larger FAZ perimeter. Regarding Aix, patients with Aix ≥ 14.745% had increased odds of having DME and increased macular thickness at the superior and temporal (perifoveal and parafoveal) quadrants. Additionally, patients with DM were found to have significantly increased PBR5-25 compared to controls without DM. To our knowledge, this is the first study using the PBR5-25 as an indirect assessment of endothelial glycocalyx integrity and examining its association with OCT and OCT-a parameters in patients with DM type 2.

The endothelial glycocalyx plays a critical role in vascular health and it is considered an important indicator of capillary blood flow, due to its impact on viscosity, which increases exponentially in vessels with narrow lumens [32,33]. Conditions such as DM and atherosclerosis can lead to degradation of the glycocalyx and consequent endothelial damage, resulting in increased vascular permeability, inflammation, and impaired capillary blood flow [34,35]. In fact, endothelial dysfunction is a hallmark of DM and is characterized by an impaired ability of the endothelium to regulate vascular tone and blood flow and maintain homeostasis. Hyperglycemia reduces nitric oxide (NO) production, leading to vasoconstriction, increased vascular resistance, and higher blood pressure [13]. Of note, NO is further reduced by the production of reactive oxygen species (ROS), which cause oxidative damage to endothelial cells [36].

In our study, we confirmed the well-known association between endothelial damage and DM, since patients with DM were found to have significantly increased PBR5-25 compared to controls without DM. Moreover, the decrease in peripapillary VD at the superior quadrant in patients with increased PBR5-25 and thus impaired endothelium could be attributed to the narrowing of the retinal capillaries due to structural changes in DM, such as the thickening of the capillaries’ basement membrane, which in turn ends up to non-perfusion. Previous studies have also found that diabetic patients, even without retinopathy, have reduced peripapillary VD compared to healthy individuals, a reduction that is associated with the duration and severity of DM [37,38,39]. However, no other study has tried to investigate the potential association of the structural findings on OCT and OCT-A with endothelial function, which was found in this study.

Another interesting finding of our study was the association between increased PWV and enlargement of FAZ area, as well as greater FAZ perimeter. It is worthwhile noting that increased PWV and consequent arterial stiffness are important cardiovascular complications associated with DM [15,40,41,42]. Both are critical markers of cardiovascular risk and are linked to the pathophysiological changes induced by chronic hyperglycemia [38,40]. The enlargement of the FAZ area and the great FAZ perimeter in OCT-A in patients with DM are indicators of retinal non-perfusion, demonstrating reduced blood flow and oxygenation of the central retina, which progressively contributes to the degeneration of retinal layers [43,44,45]. As the disease progresses, the loss of capillary networks around the fovea exacerbates FAZ enlargement, commonly correlating with poorer visual outcomes [18,46]. Interestingly, since an enlarged FAZ is associated with more severe stages of DR, monitoring of the FAZ area can help in assessing disease progression and the effectiveness of treatments [47].

In addition, the association between the enlargement of the FAZ area and the increased PWV suggests that patients with an enlarged FAZ area are more prone to cardiovascular complications, based on the fact that increased PWV is a hallmark of cardiovascular risk [41,43]. Studies have shown that retinal microvascular abnormalities, including changes in the FAZ, correlate with an increased risk of cardiovascular events, such as myocardial infarction and stroke [48]. It is worthwhile mentioning that the role of OCT-A has gained attention as a potential biomarker for systemic diseases, including cardiovascular risk [22,49,50,51]. This interest derives from the shared microvascular pathology between retinal and cardiovascular systems since both vascular beds are affected by endothelial function [22]. Therefore, FAZ measurement in OCT-A could serve as a non-invasive and simple indicator of screening in patients with DM type 2, identifying a vulnerable population for cardiovascular risk, although this should be interpreted with caution.

We also found an association between increased Aix, which is a measure of arterial stiffness and the presence of DME, as well as an increase in retinal thickness in both superior and temporal macular quadrants. In fact, in DM, chronic hyperglycemia induces endothelial damage and increased arterial stiffness, as well as high Aix. This stiffening of arteries can contribute to increased pulsatile stress on retinal vessels, exacerbating microvascular damage and promoting the development of DR [52], since elevated Aix reflects increased central pulse pressure, which can lead to greater hemodynamic stress on the retinal microvasculature [53], although Ogawa et al. reported contradictory results, showing that DR was associated with PWV but not Aix [54]. Of note, recent research suggests a correlation between increased Aix and the presence and severity of DR [10,55]. Lim et al. also described an increase in Aix in patients with DME, consistent with our findings, pointing out Aix as a significant predictor of clinically significant macular edema and vision-threatening DR [10]. This observation could be explained by the disruption of the inner blood–retinal barrier due to endothelial damage and arterial stiffness, as is expressed by the increase in Aix, leading to vascular hyperpermeability and therefore to macular edema and increased macular thickness, as was found in our study.

Potential limitations of this study pertain to its cross-sectional design, prevailing from establishing a causal relationship between examined parameters, as well as the relatively small study sample. Regarding the endothelial glycocalyx, there is no technique to quantify its presence in the retinal microcirculation in vivo; therefore, it was approximated from the sublingual microcirculation. Lastly, all limitations related to quantitative OCT-A imaging, especially its moderate repeatability, also apply to our study [56].

In conclusion, markers of endothelial damage and impaired vessel wall compliance were associated with structural and microvascular retinal alterations in patients with DM. Specifically, the most important finding of this study was that enlargement of FAZ area and greater FAZ perimeter were associated with increased PWV and therefore increased arterial stiffness, while increased macular thickness (macular edema) was related to increased Aix. Additionally, peripapillary VD was associated with endothelial damage in patients with DM. Based on these observations, regular comprehensive eye examinations for patients with DM, including assessments of peripapillary VD and overall retinal microvasculature by OCT-A, are useful and could serve as a surrogate biomarker of detecting indirectly arterial stiffness and endothelial dysfunction in patients with DM type 2. The findings of this study shed further light on DM pathophysiology, suggesting that the extent of DM-induced damage is not independent of the patient’s local and systemic vascular profile. However, since diabetic patients are patients with many comorbidities and complexities, the cardiovascular assessment by specialist physicians should not be omitted in these patients, emphasizing the importance of close monitoring for early detection of macro- and micro-vascular complications, as well as early intervention. Further large-scale prospective studies are needed to confirm our findings in a more diverse population and are necessary to establish whether these associations, aside from the pathophysiological perspective, are strong enough to predict clinical outcomes. 

## Figures and Tables

**Table 1:** Demographic and clinical characteristics of the study sample (*n* = 65 patients).

Age (years, mean ± SD)	66.6 ± 8.7
Gender (*n*, %) *Male* *Female*	38 (58.5%) 27 (41.5%)
Smoking (*n*, %)	22 (33.8%)
DM duration (years, mean ± SD)	16.4 ± 11.2
HbA1c (%, mean ± SD)	7.2 ± 1.1
Total cholesterol (mg/dL, mean ± SD)	187.3 ± 42.9
LDL (mg/dL, mean ± SD)	113.5 ± 31.4
HDL (mg/dL, mean ± SD)	43.8 ± 14.2
Triglycerides (mg/dL, mean ± SD)	152.4 ± 63.8
Hypertension (*n*, %)	48 (73.8%)
Dyslipidemia (*n*, %)	52 (80.0%)
Neuropathy (*n*, %)	15 (23.1%)
Nephropathy (*n*, %)	6 (9.2%)
Diabetic macular edema (*n*, %)	28 (43.1%)
Diabetic retinopathy (*n*, %) *No* *Mild non-proliferative* *Moderate non-proliferative* *Severe non-proliferative* *Proliferative*	20 (30.8%) 3 (4.6%) 14 (21.5%) 23 (35.4%) 5 (7.7%)
Visual acuity (decimal, mean ± SD)	0.44 ± 0.19
Intraocular pressure (mmHg, mean ± SD)	13.4 ± 3.1
Lens status (*n*, %) *Phakic* *Pseudophakic*	42 (64.6%) 23 (35.4%)

**Table 2:** Systemic vascular metrics in our study sample (*n* = 65 patients).

**Glycocalyx PBR (mean ± SD**)	
5–25 μm	2.20 ± 0.26
5–9 μm	1.28 ± 0.23
10–19 μm	2.33 ± 0.28
20–25 μm	2.74 ± 0.43
**Vessel wall compliance**	
cSBP (mean ± SD, mmHg)	130.4 ± 20.6
cDBP (mean ± SD, mmHg)	77.6 ± 10.1
PP (mean ± SD, mmHg)	50.7 ± 26.4
Aix (mean ± SD, %)	16.3 ± 23.6
PWV (mean ± SD, m/s)	13.5 ± 4.9

Aix: augmentation index; cDBP: central diastolic blood pressure; cSBP: central systolic blood pressure; IQR: interquartile range; PBR: perfused boundary region; PP: pulse pressure; PWV: pulse wave velocity; SD: standard deviation.

**Table 3:** Retinal parameters in our study sample (*n* = 65).

**Macular Thickness (μm)**	
Central subfield thickness (mean ± SD)	257.4 ± 36.2
Parafoveal superior (mean ± SD)	278.5 ± 31.3
Parafoveal inferior (mean ± SD)	273.0 ± 29.9
Parafoveal nasal (mean ± SD)	290.5 ± 31.8
Parafoveal temporal (mean ± SD)	280.6 ± 46.2
Perifoveal superior (mean ± SD)	316.7 ± 34.7
Perifoveal inferior (mean ± SD)	316.9 ± 34.2
Perifoveal nasal (mean ± SD)	313.3 ± 31.6
Perifoveal temporal (mean ± SD)	318.4 ± 43.6
**RNFL thickness (μm)**	
Average (mean ± SD)	100.1 ± 12.9
Superior (mean ± SD)	120.2 ± 23.4
Inferior (mean ± SD)	125.6 ± 18.0
Nasal (mean ± SD)	75.9 ± 14.4
Temporal (mean ± SD)	78.8 ± 17.3
**Macular OCT-A**	
VD, Central (%, mean ± SD)	18.9 ± 5.5
VD, Superior (%, mean ± SD)	41.2 ± 4.73
VD, Inferior (%, mean ± SD)	40.8 ± 4.6
VD, Nasal (%, mean ± SD)	41.5 ± 5.4
VD, Temporal (%, mean ± SD)	42.3 ± 4.6
FAZ area (mm^2^, mean ± SD)	0.343± 0.146
FAZ perimeter (mm, mean ± SD)	2.723 ± 0.569
FAZ circularity index (mean ± SD)	0.560 ± 0.079
**Peripapillary OCT-A**	
VD, Average (%, mean ± SD)	41.5 ± 3.19
VD, Superior (%, mean ± SD)	46.9 ± 4.6
VD, Inferior (%, mean ± SD)	47.9 ± 4.35
VD, Nasal (%, mean ± SD)	40.9 ± 5.31
VD, Temporal (%, mean ± SD)	44.2 ± 3.79

FAZ: foveal avascular zone; IQR: interquartile range; mVD: macular vessel density; OCT-A: optical coherence tomography angiography; pVD: peripapillary vessel density; RNFL: retinal nerve fiber layer; SD: standard deviation.

**Table 4:** Results of a series of logistic regression models examining the associations between the increased diameter of the perfused boundary region of the sublingual arterial microvessel with a diameter that ranged from 5 to 25 μm (≥2.19 versus <2.19) and eye imaging variables.

Eye Imaging (Dependent Variables)	Univariate OR (95% CI)	*p*-Value	Multivariate OR (95% CI), Adjusting for Gender, Age *, DM Duration *	*p*-Value
Diabetic Retinopathy (Yes vs. No)	1.53 (0.53–4.41)	0.433	2.06 (0.63–6.67)	0.230
Diabetic Macular Edema (Yes vs. No)	1.38 (0.52–3.69)	0.515	1.33 (0.49–3.62)	0.575
**OCT parameters**				
Central Subfield Thickness (≥271 μm vs. <271 μm)	1.54 (0.58–4.09)	0.389	1.43 (0.53–3.91)	0.481
Perifoveal Superior Thickness (≥318 μm vs. <318 μm)	1.52 (0.57–4.06)	0.400	1.80 (0.64–5.03)	0.265
Perifoveal Inferior Thickness (≥314 μm vs. <314 μm)	1.18 (0.44–3.14)	0.746	1.30 (0.47–3.56)	0.616
Perifoveal Nasal Thickness (≥319 μm vs. <319 μm)	1.20 (0.45–3.18)	0.714	1.33 (0.49–3.63)	0.579
Perifoveal Temporal Thickness (≥322 μm vs. <322 μm)	1.98 (0.74–5.31)	0.176	2.17 (0.76–6.18)	0.145
Parafoveal Superior Thickness (≥269 μm vs. <269 μm)	1.05 (0.40–2.79)	0.915	1.16 (0.42–3.16)	0.778
Parafoveal Inferior Thickness (≥266 μm vs. <266 μm)	1.54 (0.58–4.09)	0.389	1.55 (0.57–4.26)	0.391
Parafoveal Nasal Thickness (≥283 μm vs. <283 μm)	1.54 (0.58–4.09)	0.389	1.53 (0.63–4.76)	0.290
Parafoveal Temporal Thickness (≥265 μm vs. <265 μm)	1.20 (0.45–3.18)	0.714	1.24 (0.46–3.35)	0.677
**Macular OCT-A parameters**				
FAZ area (≥0.304 mm^2^ vs. <0.304 mm^2^)	1.66 (0.62–4.46)	0.318	1.62 (0.59–4.43)	0.344
FAZ perimeter (≥2.601 mm vs. <2.601 mm)	1.00 (0.37–2.67)	> 0.999	1.04 (0.38–2.84)	0.943
FAZ circularity (≥0.568 vs. <0.568)	1.29 (0.48–3.44)	0.617	1.15 (0.40–3.31)	0.799
Vessel Density Central (≥19.82% vs. <19.82%)	0.60 (0.22–1.62)	0.318	0.59 (0.21–1.62)	0.303
Vessel Density Superior (≥41.38% vs. <41.38%)	1.00 (0.37–2.67)	> 0.999	1.06 (0.39–2.91)	0.905
Vessel Density Inferior (≥41.05% vs. <41.05%)	1.29 (0.48–3.44)	0.617	1.20 (0.40–3.61)	0.741
Vessel Density Nasal (≥42.19% vs. <42.19%)	0.78 (0.29–2.08)	0.617	0.76 (0.27–2.09)	0.590
Vessel Density Temporal (≥41.98% vs. <41.98%)	0.78 (0.29–2.08)	0.617	0.78 (0.28–2.18)	0.644
**OCT optic nerve parameters**				
RNFL Average Thickness (≥101 μm vs. <101 μm)	1.20 (0.45–3.18)	0.714	1.19 (0.44–3.25)	0.734
RNFL Superior Thickness (≥124 μm vs. <124 μm)	1.20 (0.45–3.18)	0.714	1.28 (0.47–3.48)	0.631
RNFL Inferior Thickness (≥126 μm vs. <126 μm)	0.73 (0.28–1.94)	0.531	0.79 (0.29–2.15)	0.640
RNFL Nasal Thickness (≥73 μm vs. <73 μm)	1.54 (0.58–4.09)	0.389	1.62 (0.58–4.53)	0.354
RNFL Temporal Thickness (≥77 μm vs. <77 μm)	2.56 (0.94–6.96)	0.066	2.61 (0.92–7.40)	0.071
**OCT-A optic nerve parameters**				
Vessel Density Superior (≥47.74% vs. <47.74%)	0.34 (0.12–0.93)	**0.037**	0.37 (0.12–1.12)	0.079
Vessel Density Inferior (≥48.17% vs. <48.17%)	0.57 (0.21–1.52)	0.263	0.52 (0.19–1.48)	0.223
Vessel Density Nasal (≥40.9% vs. <40.9%)	0.94 (0.35–2.48)	0.897	0.99 (0.36–2.68)	0.997
Vessel Density Temporal (≥43.9% vs. <43.9%)	0.44 (0.16–1.20)	0.108	0.47 (0.16–1.35)	0.161

* Age and DM duration were entered into the models as binary variables (≥median and <median). Specifically, the median value of age was 68 years, and for DM, the duration was 15 years.

**Table 5:** Results of a series of logistic regression models examining the associations between increased pulse wave velocity (≥13.7 m/s versus <13.7 m/s) and eye imaging variables.

Eye Imaging (Dependent Variables)	Univariate OR (95% CI)	*p*-Value	Multivariate OR (95% CI), Adjusting for Gender, Age *, DM Duration *	*p*-Value
Diabetic Retinopathy (Yes vs. No)	2.79 (0.93–8.33)	0.067	3.10 (0.94–10.18)	0.063
Diabetic Macular Edema (Yes vs. No)	1.78 (0.66–4.78)	0.252	1.74 (0.64–4.70)	0.277
**OCT parameters**				
Central Subfield Thickness (≥271 μm vs. <271 μm)	0.94 (0.35–2.48)	0.897	0.89 (0.33–2.41)	0.824
Perifoveal Superior Thickness (≥318 μm vs. <318 μm)	1.96 (0.73–5.28)	0.182	2.01 (0.73–5.54)	0.179
Perifoveal Inferior Thickness (≥314 μm vs. <314 μm)	0.71 (0.26–1.91)	0.498	0.72 (0.26–1.94)	0.511
Perifoveal Nasal Thickness (≥319 μm vs. <319 μm)	0.44 (0.16–1.20)	0.108	0.43 (0.16–1.18)	0.100
Perifoveal Temporal Thickness (≥322 μm vs. <322 μm)	1.20 (0.45–3.18)	0.714	1.14 (0.42–3.11)	0.804
Parafoveal Superior Thickness (≥269 μm vs. <269 μm)	0.82 (0.31–2.19)	0.697	0.82 (0.31–2.22)	0.701
Parafoveal Inferior Thickness (≥266 μm vs. <266 μm)	0.57 (0.21–1.52)	0.263	0.54 (0.20–1.49)	0.235
Parafoveal Nasal Thickness (≥283 μm vs. <283 μm)	0.73 (0.28–1.94)	0.531	0.73 (0.27–1.96)	0.533
Parafoveal Temporal Thickness (≥265 μm vs. <265 μm)	0.94 (0.35–2.48)	0.897	0.92 (0.35–2.47)	0.875
**Macular OCT-A parameters**				
FAZ area (≥0.304 mm^2^ vs. <0.304 mm^2^)	2.79 (1.01–7.70)	**0.048**	2.88 (1.03–8.04)	**0.044**
FAZ perimeter (≥2.601 mm vs. <2.601 mm)	2.79 (1.01–7.70)	**0.048**	2.82 (1.01–7.89)	**0.048**
FAZ circularity (≥0.568 vs. <0.568)	1.00 (0.37–2.67)	> 0.999	1.04 (0.36–2.96)	0.947
Vessel Density Central (≥19.82% vs. <19.82%)	0.47 (0.17–1.27)	0.135	0.47 (0.17–1.29)	0.143
Vessel Density Superior (≥41.38% vs. <41.38%)	0.78 (0.29–2.08)	0.617	0.79 (0.29–2.14)	0.639
Vessel Density Inferior (≥41.05% vs. <41.05%)	1.00 (0.37–2.67)	>0.999	1.07 (0.36–3.16)	0.909
Vessel Density Nasal (≥42.19% vs. <42.19%)	0.36 (0.13–0.99)	**0.048**	0.36 (0.13–1.02)	0.054
Vessel Density Temporal (≥41.98% vs. <41.98%)	0.47 (0.17–1.27)	0.135	0.46 (0.16–1.30)	0.143
**OCT optic nerve parameters**				
RNFL Average Thickness (≥101 μm vs. <101 μm)	0.73 (0.28–1.94)	0.531	0.77 (0.28–2.07)	0.598
RNFL Superior Thickness (≥124 μm vs. <124 μm)	1.20 (0.45–3.18)	0.714	1.26 (0.47–3.40)	0.643
RNFL Inferior Thickness (≥126 μm vs. <126 μm)	0.57 (0.21–1.52)	0.263	0.59 (0.22–1.62)	0.307
RNFL Nasal Thickness (≥73 μm vs. <73 μm)	1.54 (0.58–4.09)	0.389	1.69 (0.61–4.67)	0.312
RNFL Temporal Thickness (≥77 μm vs. <77 μm)	0.73 (0.28–1.94)	0.531	0.69 (0.25–1.89)	0.473
**OCT-A optic nerve parameters**				
Vessel Density Superior (≥47.74% vs. <47.74%)	0.94 (0.35–2.48)	0.897	1.03 (0.35–3.01)	0.963
Vessel Density Inferior (≥48.17% vs. <48.17%)	0.73 (0.28–1.94)	0.531	0.75 (0.28–2.06)	0.579
Vessel Density Nasal (≥40.9% vs. <40.9%)	1.20 (0.45–3.18)	0.714	1.26 (0.47–3.40)	0.643
Vessel Density Temporal (≥43.9% vs. <43.9%)	1.54 (0.58–4.09)	0.389	1.54 (0.55–4.36)	0.413

* Age and DM duration were entered into the models as binary variables (≥median and <median). Specifically, the median value of age was 68 years, and for DM, the duration was 15 years.

**Table 6:** Results of a series of logistic regression models examining the associations between increased augmentation index (≥14.745 versus <14.745) and eye imaging variables.

Eye Imaging (Dependent Variables)	Univariate OR (95% CI)	*p*-Value	Multivariate OR (95% CI), Adjusting for Gender, Age *, DM Duration *	*p*-Value
Diabetic Retinopathy (Yes vs. No)	1.80 (0.62–5.27)	0.283	3.43 (0.92–12.83)	0.067
Diabetic Macular Edema (Yes vs. No)	2.45 (0.89–6.74)	0.081	3.05 (1.00–9.34)	**0.050**
**OCT parameters**				
Central Subfield Thickness (≥271 μm vs. <271 μm)	2.14 (0.79–5.79)	0.136	2.54 (0.84–7.65)	0.099
Perifoveal Superior Thickness (≥318 μm vs. <318 μm)	2.14 (0.79–5.82)	0.135	3.37 (1.07–10.60)	**0.038**
Perifoveal Inferior Thickness (≥314 μm vs. <314 μm)	1.91 (0.70–5.22)	0.208	2.47 (0.81–7.54)	0.111
Perifoveal Nasal Thickness (≥319 μm vs. <319 μm)	0.78 (0.29–2.08)	0.617	0.88 (0.31–2.53)	0.819
Perifoveal Temporal Thickness (≥322 μm vs. <322 μm)	2.14 (0.79–5.79)	0.136	3.37 (1.04–10.90)	**0.043**
Parafoveal Superior Thickness (≥269 μm vs. <269 μm)	2.44 (0.89–6.65)	0.082	3.47 (1.11–10.78)	**0.032**
Parafoveal Inferior Thickness (≥266 μm vs. <266 μm)	1.65 (0.62–4.44)	0.319	1.78 (0.61–5.24)	0.294
Parafoveal Nasal Thickness (≥283 μm vs. <283 μm)	1.00 (0.38–2.66)	>0.999	1.15 (0.40–3.30)	0.790
Parafoveal Temporal Thickness (≥265 μm vs. <265 μm)	2.78 (1.01–7.64)	**0.048**	3.41 (1.11–10.49)	**0.032**
**Macular OCT-A parameters**				
FAZ area (≥0.304 mm^2^ vs. <0.304 mm^2^)	0.64 (0.24–1.73)	0.380	0.55 (0.19–1.63)	0.283
FAZ perimeter (≥2.601 mm vs. <2.601 mm)	0.56 (0.21–1.52)	0.258	0.64 (0.22–1.86)	0.408
FAZ circularity (≥0.568 vs. <0.568)	1.38 (0.52–3.71)	0.528	1.28 (0.42–3.92)	0.670
Vessel Density Central (≥19.82% vs. <19.82%)	2.02 (0.74–5.52)	0.168	1.90 (0.65–5.54)	0.239
Vessel Density Superior (≥41.38% vs. <41.38%)	1.38 (0.51–3.71)	0.528	1.49 (0.51–4.35)	0.467
Vessel Density Inferior (≥41.05% vs. <41.05%)	1.78 (0.66–4.83)	0.258	1.88 (0.57–6.18)	0.297
Vessel Density Nasal (≥42.19% vs. <42.19%)	0.83 (0.31–2.22)	0.707	0.62 (0.21–1.86)	0.393
Vessel Density Temporal (≥41.98% vs. <41.98%)	0.83 (0.31–2.22)	0.707	0.75 (0.25–2.25)	0.612
**OCT optic nerve parameters**				
RNFL Average Thickness (≥101 μm vs. <101 μm)	1.28 (0.48–3.43)	0.617	1.03 (0.36–2.98)	0.950
RNFL Superior Thickness (≥124 μm vs. <124 μm)	0.60 (0.23–1.62)	0.319	0.51 (0.17–1.50)	0.221
RNFL Inferior Thickness (≥126 μm vs. <126 μm)	0.69 (0.26–1.84)	0.454	0.58 (0.19–1.72)	0.324
RNFL Nasal Thickness (≥73 μm vs. <73 μm)	1.65 (0.62–4.44)	0.319	1.54 (0.53–4.51)	0.431
RNFL Temporal Thickness (≥77 μm vs. <77 μm)	1.28 (0.48–3.43)	0.617	1.29 (0.44–3.78)	0.648
**OCT-A optic nerve parameters**				
Vessel Density Superior (≥47.74% vs. <47.74%)	0.47 (0.17–1.27)	0.136	0.43 (0.13–1.40)	0.161
Vessel Density Inferior (≥48.17% vs. <48.17%)	1.28 (0.48–3.43)	0.617	1.19 (0.40–3.47)	0.757
Vessel Density Nasal (≥40.9% vs. <40.9%)	1.88 (0.70–5.07)	0.213	1.96 (0.67–5.73)	0.219
Vessel Density Temporal (≥43.9% vs. <43.9%)	0.60 (0.23–1.62)	0.319	0.90 (0.30–2.64)	0.843

* Age and DM duration were entered into the models as binary variables (≥median and <median). Specifically, the median value of age was 68 years and for DM duration it was 15 years.

## Data Availability

The original contributions presented in the study are included in the article; further inquiries can be directed to the corresponding authors.
